# Bony Stroke on Rheumatoid Arthritis: A Rare Entity or a Missed Diagnosis?

**DOI:** 10.7759/cureus.74067

**Published:** 2024-11-20

**Authors:** Sofia Vedor, Sara de Carvalho, Gonçalo Alves, Duarte Vieira

**Affiliations:** 1 Neuroradiology Department, Unidade Local de Saúde de São João, Porto, PRT

**Keywords:** bony stroke, cranial settling, osteovascular conflict, rheumatoid arthritis, vertebrobasilar insufficiency (vbi)

## Abstract

Bone or cartilage anomalies affecting the arteries supplying the brain can be a structural cause of ischemic stroke. Due to their rarity, there is currently no standardized approach for evaluating and treating these so-called bony strokes. We present a case of a 79-year-old woman with a history of cranial settling due to rheumatoid arthritis (RA) and moderate disability, who presented with insidious dizziness and gait disturbances over three weeks. Clinical examination unveiled impaired cognition and discrete left-side dysmetria. Initial head computed tomography (CT) showed signs of a recent stroke within the left posterior inferior cerebellar artery (PICA) territory, with chronic infarctions in the contralateral cerebellum. CT angiography (CTA) identified subocclusive stenosis in the V3 segment of the left vertebral artery (VA), attributed to cranial settling progression. An ultrasonographic study revealed increased flow resistance in both V2 segments, with downstream normalization at the V4 level. A diagnosis of bony stroke was established. This case underscores the significance of considering bone abnormalities as structural causes of ischemic stroke, especially in recurrent cryptogenic events within a dependent territory. This consideration is particularly critical for patients with cognitive impairment, which can obscure accurate diagnoses and delay treatment.

## Introduction

Bone abnormalities can act as structural causes of ischemic stroke, particularly in cases of recurrent cryptogenic events affecting a single vascular territory. These "bony strokes" occur when skeletal deformities compress or obstruct blood flow in nearby arteries, leading to ischemic events [1-3].

Rheumatoid arthritis (RA) is a chronic inflammatory condition commonly affecting the cervical spine. It leads to degeneration, instability, and, in rare cases, cranial settling, a downward displacement of the skull base. These changes can lead to symptoms of vertebrobasilar insufficiency, caused by the dynamic compression of the dominant vertebral artery (VA), which disrupts blood flow to the posterior brain circulation [2-5].

Despite its significance, the diagnosis of bony strokes remains challenging due to their rarity and the lack of standardized diagnostic protocols. Understanding these conditions is crucial to prevent underdiagnosis or misdiagnosis, particularly in RA patients with unexplained ischemic events.

This report presents a case of RA with cervical spine involvement and ischemic stroke secondary to vertebrobasilar insufficiency in the context of cranial settling. We aim to explore the clinical presentation and pathogenesis of this condition, emphasizing critical imaging correlations between bone abnormalities and ischemic strokes in RA patients.

## Case presentation

A 79-year-old woman with a long history of seropositive RA, cranial settling, and moderate disability presented with an insidious onset of dizziness and gait disturbances over the previous three weeks. Additionally, she reported two episodes of vomiting within the 24 hours before admission. There was no history of fever and headache or records of cervical trauma.

**Figure 1:**
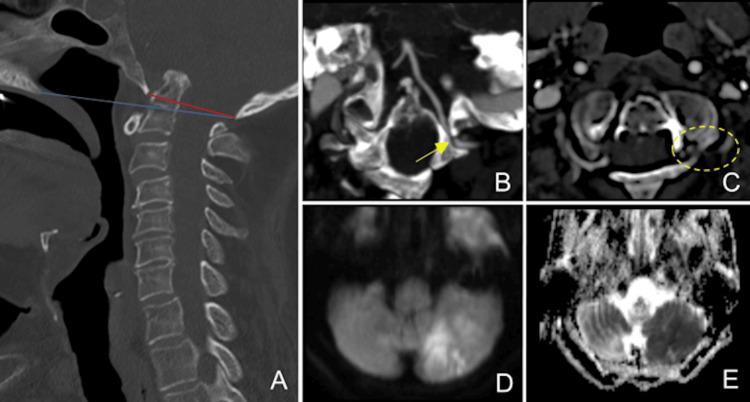
Head and Neck CT Angiography and Brain MRI. (A) CT angiography of the craniovertebral junction in the bone algorithm shows an upward displacement of the dens axis (C2) crossing the McRae (red) and Chamberlain (blue) lines, caused by the collapse of local supporting structures. (B and C) Tissue algorithm images reveal subocclusive stenosis in the distal V3 segment of the left vertebral artery (yellow arrow and dot circle), due to a reduction in space between the occipital condyle and the lateral mass of the atlas. These images illustrate structural abnormalities associated with cranial settling, leading to subocclusive stenosis in the left vertebral artery, a critical finding for bony stroke diagnosis. (D and E) Brain MRI depicts cortico-subcortical hyperintensity at the left cerebellar hemisphere on DWI (D) with diffusion restriction on the ADC map (E), confirming a recent ischemic lesion in the left PICA territory, which correlates with the patient's clinical symptoms. CT, computed tomography; MRI, magnetic resonance imaging; DWI, diffusion-weighted imaging; ADC, apparent diffusion coefficient; PICA, posterior inferior cerebellar artery

Upon admission to the emergency department, the patient exhibited hypotension and tachycardia, alongside cognitive impairment and subtle left-side dysmetria. Initial head computed tomography (CT) revealed a cortico-subcortical hypodensity at the inferior left cerebellar hemisphere, with corresponding T2/fluid-attenuated inversion recovery (FLAIR) hyperintensity and diffusion restriction on brain magnetic resonance imaging (MRI), suggesting a recent ischemic lesion in the left posterior inferior cerebellar artery (PICA) territory (Figure 1D, 1E). Chronic infarctions were also identified within the right cerebellar hemisphere, confirming prior posterior circulation ischemic episodes. There were no signs of recent bleeding in the intracranial compartment. CT angiography (CTA) revealed a subocclusive stenosis in the distal V3 segment of the left vertebral artery (VA), attributed to the progressive nature of the cranial settling (Figure 1A). Multiplanar reformations (MPR) confirmed a reduction in the space between the occipital condyle (at the lateral aspect of the foramen magnum) and the lateral mass of the atlas (Figure 1B, 1C), leading to focal osteovascular conflict.

**Figure 2:**
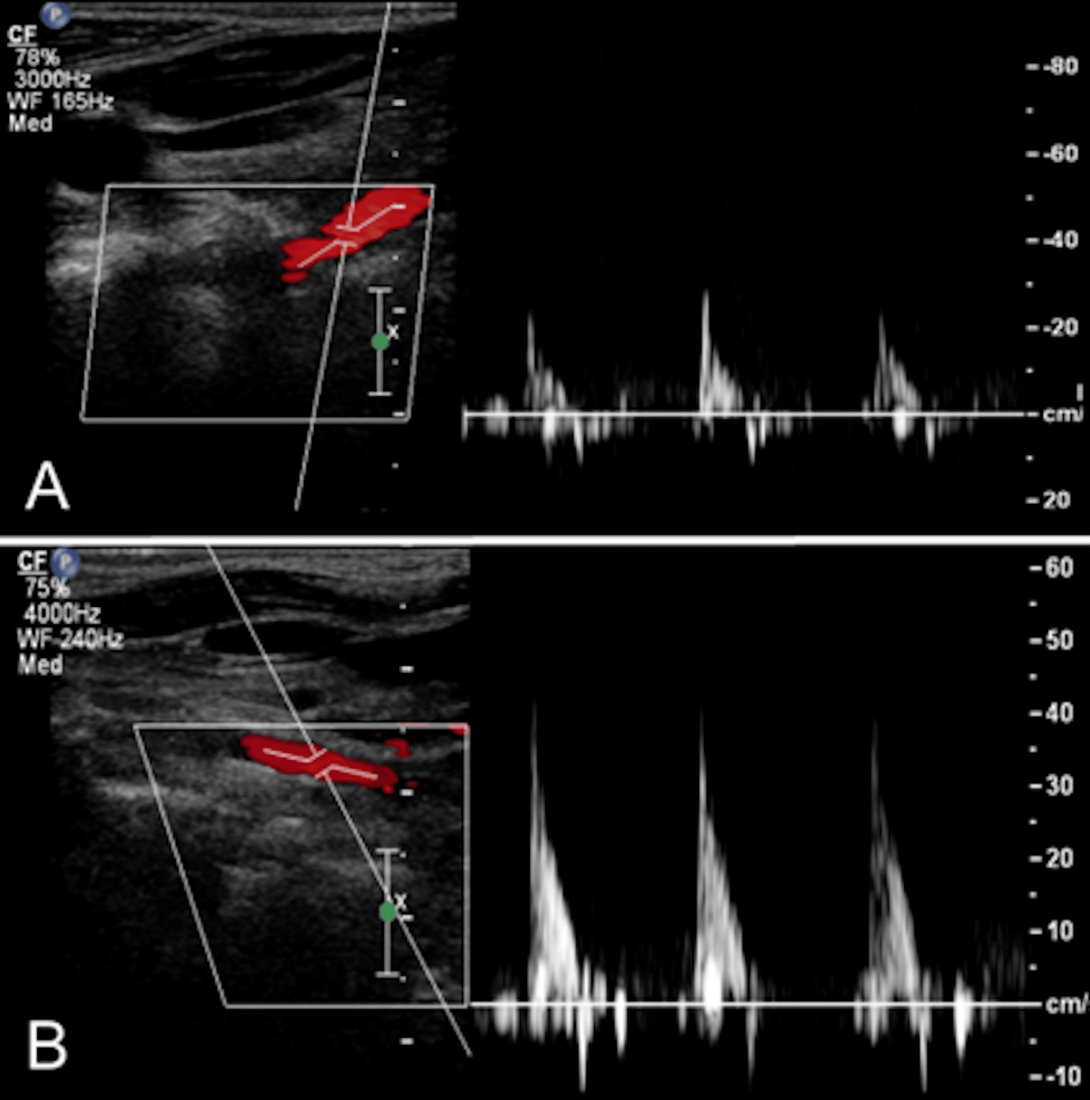
Extracranial Doppler Imaging. Spectral color Doppler ultrasound reveals a high flow resistance pattern in the V2 segment of the right (A) and left (B) vertebral arteries, with downstream normalization at the intracranial level (V4). The increased flow resistance in the vertebral arteries supports the diagnosis of vertebrobasilar insufficiency.

Congenital hypoplasia of the right VA was also noted, with an overall reduction in its diameter and a slight irregularity of the proximal V3 segment. The PICA were correctly identified on both sides. The ultrasonographic study documented high flow resistance within the V2 segments (Figure 2A, 2B), with downstream normalization at the intracranial level (V4). The transthoracic echocardiogram was unremarkable.

Following the exclusion of other potential causes, the diagnosis of bony stroke was established through a comprehensive evaluation of imaging findings. These revealed osteovascular conflict due to cranial settling on CTA and high flow resistance in the vertebral arteries on Doppler ultrasound. The presence of chronic infarctions in the contralateral posterior circulation territory further substantiated this diagnosis, pointing to a recurrent pattern of posterior circulation ischemia. The patient's clinical presentation, characterized by cognitive deterioration and disability, likely obscured a typical history of vertebrobasilar insufficiency, adding complexity to the diagnostic process. By integrating structural abnormalities, hemodynamic changes, and clinical findings, bony stroke emerged as the most plausible etiology.

At discharge, the patient exhibited exacerbated gait and balance disturbances, prompting a referral to a long-term care facility. Given the patient's disability (score of 4 on the modified Rankin Scale), the substantial burden of ischemic lesions, and multiple comorbidities, palliative care was considered appropriate. A statin therapy (atorvastatin 40 mg once daily) was initiated due to an altered lipid profile (low-density lipoprotein {LDL} cholesterol: 75 mg/dL). By the three-month follow-up, further decline was observed with progressive loss of autonomy, likely attributable to probable coexisting dementia, compounded by recurrent respiratory infections and precarious social circumstances.

## Discussion

Despite thorough diagnostic workups, approximately 25% of ischemic strokes remain unexplained, leading recent studies to focus on covert causes such as undiagnosed atrial fibrillation, non-stenosing arterial plaques, and hypercoagulability related to cancer [6]. Haertl et al. bring attention to an additional, though rarer, structural etiology: bone or cartilage anomalies instigating mechanical stress on brain-supplying arteries [1]. These structural anomalies may induce ischemic events through vessel wall damage (causing dissection and arterial embolism) or by compressing arteries and disrupting cerebral blood flow. The chronic irritation of a vessel may also result in the formation of a pseudoaneurysm, as another source of arterial embolism [6,7]. Any of these three mechanisms can occur either consistently or in relation to changes in the patient's head position. Among these conditions, the compressive affection of the VA, triggered by head rotation to the contralateral side, is known as bow hunter syndrome (BHS) [1-3,8,9]. Each of these conditions presents distinct mechanisms that can jeopardize vascular integrity, highlighting the importance of a multidisciplinary approach for accurate diagnosis and management, involving rheumatologists, neurologists, neuroradiologists, neurosurgeons, and orthopedic surgeons. However, a systematic evaluation of the various anatomical subgroups of bony strokes is currently lacking, with the majority of evidence stemming from single case reports or small case series.

RA, which affects around 1% of the population, represents the most common inflammatory disorder associated with cervical spine involvement, reported in up to 86% of cases. Men face an increased risk of advanced cervical complications, with vertical subluxation (VS), also known as basilar invagination or cranial settling, seen in approximately 15%-20% of RA patients with cervical involvement due to structural collapse around the craniovertebral junction, especially at the C1 lateral masses [4,5]. Neurological deterioration affects only about 36% of RA patients with cervical involvement, while up to 50% of those with radiographic instability remain asymptomatic, underscoring the clinico-radiological dissociation with the inherent need for careful evaluation [4].

The diagnosis of bony stroke relies on a combination of imaging modalities, including CT, MRI, and sonography of the brain-supplying vessels. The use of dynamic imaging, particularly with the patient's head in fixed rotation or reclination, has proven valuable in detecting compressive effects on these arteries during head movements [1-3,8,9].

Recognizing bony strokes is essential in clinical practice due to the high risk of stroke recurrence and the potential for targeted treatments. In cases of recurrent strokes in a single vascular territory, symptomatic anatomical anomalies in the bone or cartilage should be included in the differential diagnosis after ruling out other causes. Treatment options are diverse, encompassing conservative management, endovascular stenting, vessel occlusion, surgical bypass, and bone or cartilage removal [1].

In our case, despite a known diagnosis of RA with cranial settling, the patient's significant disability and cognitive impairment hindered a comprehensive clinical anamnesis and physical examination, masking symptoms of vertebral insufficiency and leading to delayed diagnosis and treatment, ultimately resulting in a worse prognosis. This scenario underscores a broader concern: the limited awareness among clinicians regarding the association between cranial settling and ischemic stroke.

## Conclusions

Clinicians should be aware of the rare association between cervical spine involvement in patients with RA and vertebrobasilar insufficiency, particularly when there is no clear anamnesis and the clinical suspicion is low. This case highlights the critical role of different imaging modalities in detecting structural and hemodynamic abnormalities, with significant implications for the management of these patients.

Given the rarity and heterogeneity of such clinical presentations, a multidisciplinary approach, combined with heightened vigilance and the application of precise imaging modalities, is essential to ensure early recognition and timely intervention.
